# Hammett Correlation in the Accelerated Formation of 2,3-Diphenylquinoxalines in Nebulizer Microdroplets

**DOI:** 10.3390/molecules26165077

**Published:** 2021-08-21

**Authors:** Nathan W. Fenwick, Richard Telford, Amie Saidykhan, William H. C. Martin, Richard D. Bowen

**Affiliations:** Faculty of Life Sciences, School of Chemistry and Biosciences, University of Bradford, Bradford BD7 1DP, UK; n.w.fenwick@bradford.ac.uk (N.W.F.); r.telford@bradford.ac.uk (R.T.); a.saidykhan1@bradford.ac.uk (A.S.); w.martin@bradford.ac.uk (W.H.C.M.)

**Keywords:** microdroplets, diphenylquinoxalines, condensation, competition experiments, nebulizer, Hammett relationship

## Abstract

The accelerated formation of 2,3-diphenylquinoxalines in microdroplets generated in a nebulizer has been investigated by competition experiments in which equimolar quantities of 1,2-phenylenediamine, C_6_H_4_(NH_2_)_2_, and a 4-substituted homologue, XC_6_H_3_(NH_2_)_2_ [X = F, Cl, Br, CH_3_, CH_3_O, CO_2_CH_3_, CF_3_, CN or NO_2_], or a 4,5-disubstituted homologue, X_2_C_6_H_2_(NH_2_)_2_ [X = F, Cl, Br, or CH_3_], compete to condense with benzil, (C_6_H_5_CO)_2_. Electron-donating substituents (X = CH_3_ and CH_3_O) accelerate the reaction; in contrast, electron-attracting substituents (X = F, Cl, Br and particularly CO_2_CH_3_, CN, CF_3_ and NO_2_) retard it. A structure–reactivity relationship in the form of a Hammett correlation has been found by analyzing the ratio of 2,3-diphenylquinoxaline and the corresponding substituted-2,3-diphenylquinoxaline, giving a ρ value of −0.96, thus confirming that the electron density in the aromatic ring of the phenylenediamine component is reduced in the rate-limiting step in this accelerated condensation. This correlation shows that the phenylenediamine acts as a nucleophile in the reaction.

## 1. Introduction

One recent scientific application of microdroplets is as an environment for conducting organic synthesis at an enhanced rate in mild conditions. Thus, various reactions, particularly condensations, occur far more rapidly in the microdroplets of the nebulizer of a conventional electrospray mass spectrometer source, often at or near neutral pH and at ambient temperature, when either acid or base catalysis or heat would be required in classical alternative preparative methods [1,2,3,4,5]. The formation of C=N and C-N bonds, to produce heterocycles, in the microdroplets is especially effective, as exemplified by the Hantzsch synthesis of pyridine derivatives [4] and the facile formation of 2,3-diarylquinoxalines [6,7]. Many other scientific investigations covering a wide variety of topics have been made in microdroplets generated in a nebulizer [8,9,10,11]. A recent definitive review summarizes the scope and potential for novel research in this field [12].

One particularly relevant finding in the initial study of formation of quinoxalines (“offline”, when the nebulizer was not connected to the mass spectrometer) and protonated quinoxalines (“online”, when ions formed during the condensation could be directly analyzed by mass spectrometry) was the detection and characterization of positively charged intermediates that would be expected to be formed by nucleophilic attack of the phenylenediamine on the protonated benzil [6].

The Hammett equation is a proven method for understanding in at least a semi-quantitative manner the influence of substituents on an aromatic ring on the reaction rate [13,14,15]. Each substituent is assigned a number (the “σ constant”), obtained from experimental data, which summarizes its effects on the electron density in the ring and the consequences of these effects on the chemistry of a reactive site elsewhere in the molecule. Substituents in the 2-position normally are not considered because their electronic effects, which are of primary interest, may be obscured or overridden by steric effects. A substituent with a negative σ constant is electron releasing; consequently, it generally increases the electron density in the ring (thus potentially enhancing the nucleophilicity of another electron-donating group on that ring or reducing the electrophilicity of any electron-attracting group). On the other hand, a substituent with a positive σ constant is electron attracting; therefore, it tends to diminish the electron density in the ring (which might reduce the nucleophilicity or enhance the electrophilicity of the other group). In favorable circumstances, where there is a good correlation between the σ constant and the reactivity of the other group, a Hammett plot gives a straight line with a slope that is characteristic of the reaction (the “ρ constant” or “ρ value”), which gives insight into the nature of the process. A negative ρ value shows that the rate-limiting step involves a decrease in negative charge (or an increase in positive charge) on the other group, as would be expected if this second group acts as a nucleophile in the reaction. Conversely, a positive ρ value reveals that there is an increase in negative charge (or a reduction in positive charge) on the second group in the rate-limiting step, as would be anticipated if this second group was behaving as an electrophile.

In practice, reactions are usually studied by this powerful method by obtaining data for species with substituents with various σ constants, so as to obtain the ρ value for the process by generating the relevant Hammett plot. In some cases, it is possible to obtain appropriate information from thermodynamic data (such as equilibrium constants for dissociation of substituted benzoic acids, as in the original work [13,14]) or kinetic data (by measuring the actual rates of comparable processes). In other instances, in which obtaining “absolute” data is impossible or impractical, relative rates may be determined from the relative abundance of two possible products formed in competition experiments [16,17,18].

Certain substituents participate so extensively in π-conjugation with aromatic rings that the use of standard σ constants values does not adequately summarize their influence on the electron density distribution in the aromatic ring to which they are attached. In these cases, the use of σ+ or σ− constants often gives better Hammett plots [19,20]. A σ+ constant is appropriate in cases in which the substituent is powerfully electron-donating (corresponding to a strong +M mesomeric effect, as for example for an amino, NH_2_, group). In contrast, a σ− constant is suitable for powerfully electron-withdrawing groups such as NO_2_ that have a strong −M mesomeric effect).

**Scheme 1:**
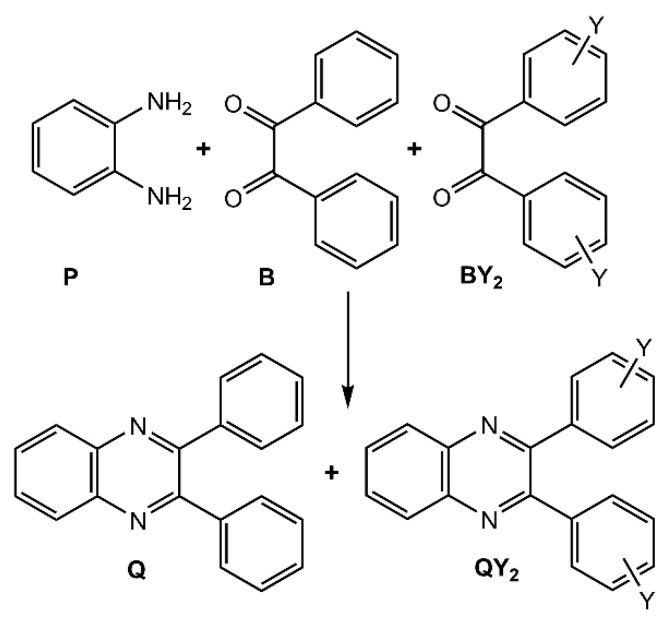
Condensation of equimolar quantities of 1,2-phenylenediamine (**P**) and benzil (**B**) or a 3,3′ or 4,4′ disubstituted benzil (**BY_2_**) to give a mixture of 2,3-diphenylquinoxaline (**Q**) and 2,3-diarylquinoxaline (**QY_2_**).

The approach of applying competition experiments to obtain relative rate data was previously [7] exploited by measuring the quantities of 2,3-diphenylquinoxaline and the corresponding 2,3-diarylquinoxaline formed when phenylenediamine reacted with equimolar quantities of benzil and a 3,3′- or 4,4′-disubstituted benzil, Scheme 1. The names of the reactants and products are abbreviated as follows: **P**, **B** and **Q** represent, respectively, 1,2-phenylenediamine, benzil and 2,3-diphenylquinoxaline; **BY_2_** and **QY_2_** represent the 3,3′- or 4,4′-disubstituted benzil and the derived 2,3-diarylquinoxaline containing substituents, Y, in the pendant aryl rings.

Previous work also established that measuring the relative abundance of **Q** and **QY_2_** from the intensity of the [M+H]^+^ signals in the positive ion electrospray, ESI+, spectra in “online” experiments (in which the effluent from the nebulizer in liquid chromatography mass spectrometry, LC-MS, analysis was introduced into the mass spectrometer) did not give satisfactory data. This disappointing outcome was attributed to the non-linear response of the detector to the [M+H]^+^ ions and difficulties in accurately calibrating this response. However, when the ratio of **Q** and **QY_2_** obtained by transmission of starting materials through the nebulizer in “offline” competition experiments was measured by liquid chromatography using the UV detector, a good Hammett correlation, with a ρ constant of 1.87 and a correlation factor (“R^2^” value) of 0.90 was obtained [7].

Closely similar results (a ρ value of 1.83 and an R^2^ value of 0.89) were obtained when the quantities of **Q** and **QY_2_** were measured by the relative areas of the peaks in the total ion chromatogram, TIC, of signals in the gas chromatography mass spectrometry, GC-MS, analysis under electron ionization, EI, conditions of the product mixture. This protocol is especially attractive because it is possible to analyze a large number of mixtures by means of the auto-sampler on the GC-MS system [7].

These results revealed that there is a substantial increase in the electron density at the carbonyl carbon atom of the benzil during the rate-limiting step in the accelerated condensation, which in turn implies that the benzil is behaving as an electrophile.

**Scheme 2:**
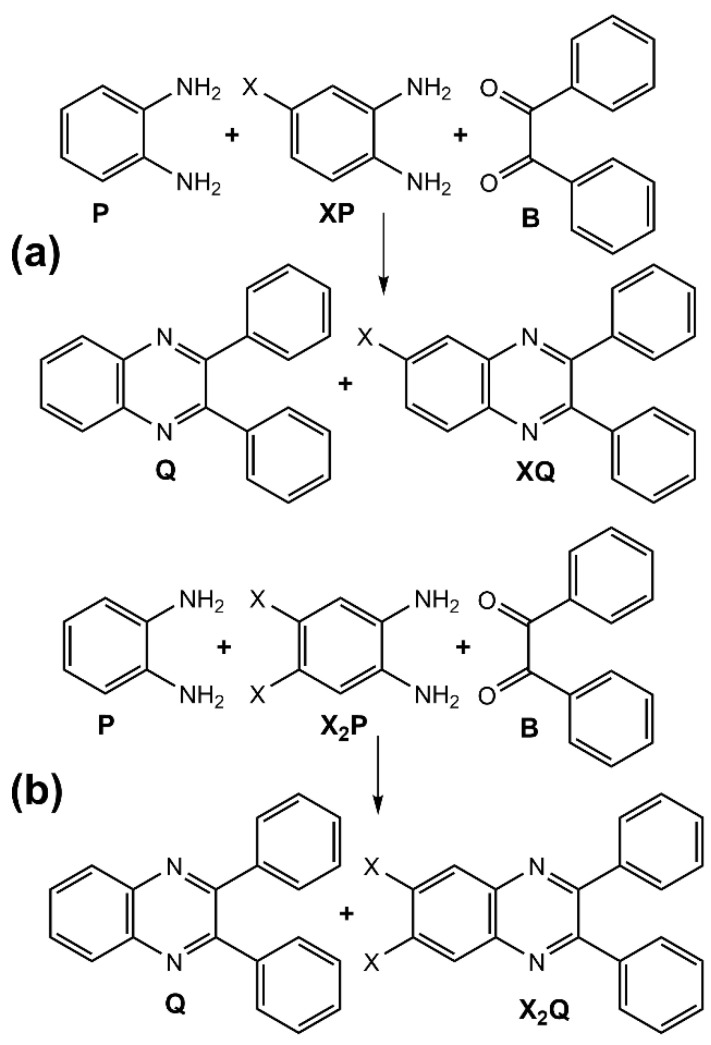
(**a**). Condensation of equimolar quantities of 1,2-phenylenediamine (**P**) and a 4-substituted-1,2-diphenylenediamine (**XP**) and benzil (**B**) to give a mixture of 2,3-diphenylquinoxaline (**Q**) and a 6-substituted-2,3-diphenylquinoxaline (**XQ**). (**b**). Condensation of equimolar quantities of 1,2-phenylenediamine (**P**) and a 4,5-disubstituted-1,2-diphenylenediamine (**X_2_P**) and benzil (**B**) to give a mixture of 2,3-diphenylquinoxaline (**Q**) and a 6,7-disubstitued-2,3-diphenylquinoxaline (**X_2_Q**).

This preliminary work suggests that further research to answer two questions is desirable. Firstly, is it possible to establish that the phenylenediamine component behaves as a nucleophile by complementary experiments, in which phenylenediamine (**P**) and a substituted phenylenediamine (abbreviated to **XP** or **X_2_P**, Scheme 2) compete to condense with benzil?

Secondly, is the use of σ values appropriate for all cases? Would it be more appropriate to use σ+ or σ− values [20], especially for substituents in the 4-position which interact strongly by π-conjugation with the reactive site by the mesomeric effect? These investigations are slightly complicated by the fact that a substituent that is in the “para” 4-position relative to one amino substituent in **XP** or **X_2_P** will be in the “meta” 3-position relative to the other amino substituent. However, in such cases, composite σ, σ+ or σ− constants may be derived [21], thus facilitating the analysis.

## 2. Results

The presence of the substituent, X, in the diphenylphenylenediamine systematically influences the ratio of **Q** and **XQ** or **Q** and **X_2_Q** formed in the nebulizer, as shown in Table 1 and Table 2, respectively.

## 3. Discussion

Firstly, the presence of an electron-donating substituent (CH_3_ or CH_3_O) in **XP** accelerates the condensation, thus favoring formation of **XQ** over **Q**, as shown by a ratio of **XQ/Q** that is greater than unity. At first sight, it is surprising that a methyl substituent has a more activating effect (**XQ/Q** ratio of 2.16) than a methoxy group (**XQ/Q** ratio of only 1.58). However, this apparent anomaly is explicable in terms of the composite σ constants that are necessary because a substituent para to one **NH_2_** group is meta relative to the other. A methyl group in either the para or meta position is electron releasing thus enhancing the nucleophilicity of the amino group(s) (σ constant of −0.07 and −0.17, respectively). In contrast, a para methoxy group is electron releasing (and activating, with a σ constant of −0.27, because of its powerful +M mesomeric effect, which overrides the −I inductive effect), but a meta methoxy group is electron-withdrawing (and deactivating, with a σ constant of 0.12, because its mesomeric effect is negligible, but its −I inductive effect is stronger because the meta position is closer to the amino group than the more distant para position). The standard composite σ constant for a methyl and methoxy group is −0.24 and −0.15, respectively.

Secondly, an electron-withdrawing and deactivating substituent (F, Cl, Br, and especially CH_3_CO_2_ CF_3_, CN, or NO_2_) has the opposite effect, reducing the nucleophilicity of the amino group(s), which leads to preferential formation of **Q**, as indicated by a **XQ/Q** ratio below unity.

Thirdly, these trends are generally reinforced in the less extensive data in the disubstituted series, as would be expected if the influence of two substituents was additive or multiplicative. Thus, the **X_2_Q/Q** ratio of 2.79 when **(CH_3_)_2_P** competes with **P** to condense with **B** is larger than that (2.16) in the corresponding competition experiments involving **CH_3_P** and **P** because there are two activating substituents in **(CH_3_)_2_P**. Conversely, when two deactivating substituents are present, the **X_2_Q/Q** ratio is smaller than when there is only one such substituent, as illustrated by the ratios of 0.196 and 0.512 when **F_2_P** and **FP**, respectively, compete with **P** to condense with **B** to form **F_2_Q** and **FQ**. However, the trend in which the second substituent reinforces the first appears to decline on progressing through the series X = F, Cl and Br.

Fourthly, although the deactivating influence of the nitro group in **O_2_NP** is greater than that of any other electron-withdrawing substituent, as revealed by the **XQ/Q** ratio of only 0.024, **O_2_NQ** is formed in the microdroplets produced in the nebulizer. This finding emphasizes the acceleration of the rate of condensations of this kind in microdroplets. Even after prolonged heating for 5–12 h at 80 °C in ethanol, there was no evidence from thin layer chromatography, tlc, of the formation of appreciable quantities of **O_2_NQ** from **O_2_NP** and **B** in conventional solution experiments. Indeed, the preparation of reference **O_2_NQ** entailed overnight heating of equimolar quantities of **O_2_NP** and **B** in ethylene glycol (or glycerol) at 120–150 °C.

**Figure 1:**
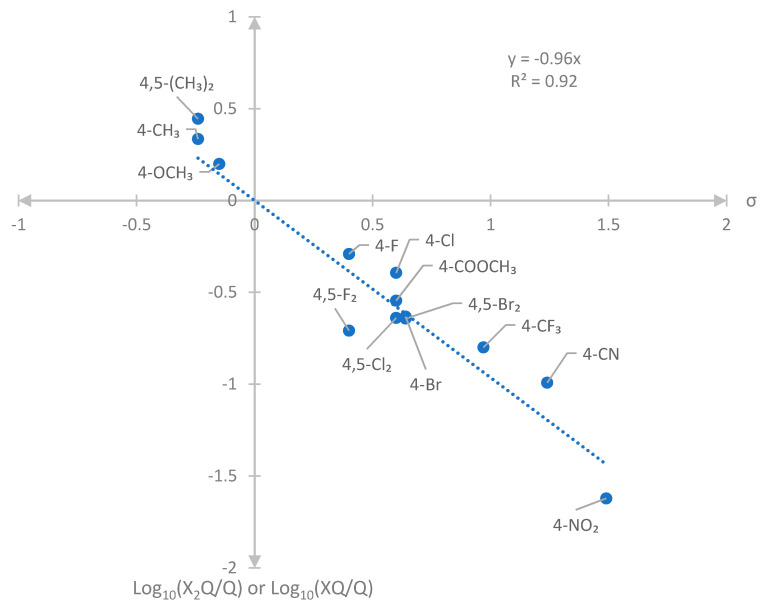
Hammett plot of **XQ/Q** or **X_2_Q/Q** ratio with standard composite σ constants.

**Figure 2:**
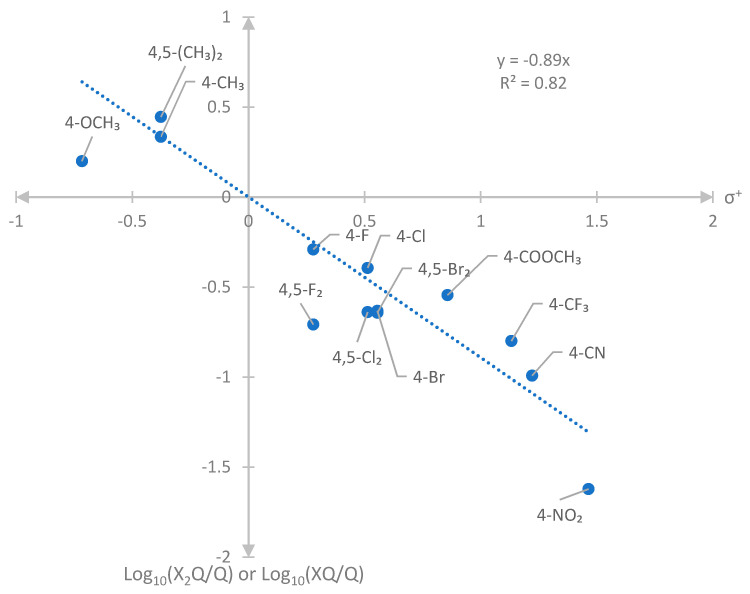
Hammett plot of **XQ/Q** or **X_2_Q/Q** ratio with standard composite σ+ constants.

**Figure 3:**
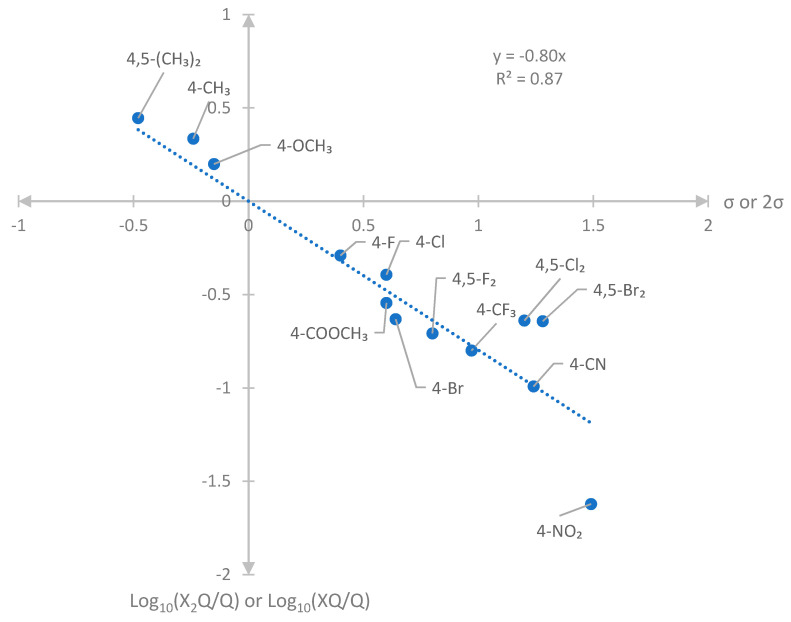
Hammett plot of **XQ/Q** ratio with standard composite σ constants and **X_2_Q/Q** ratio with doubled standard composite σ constants.

**Figure 4:**
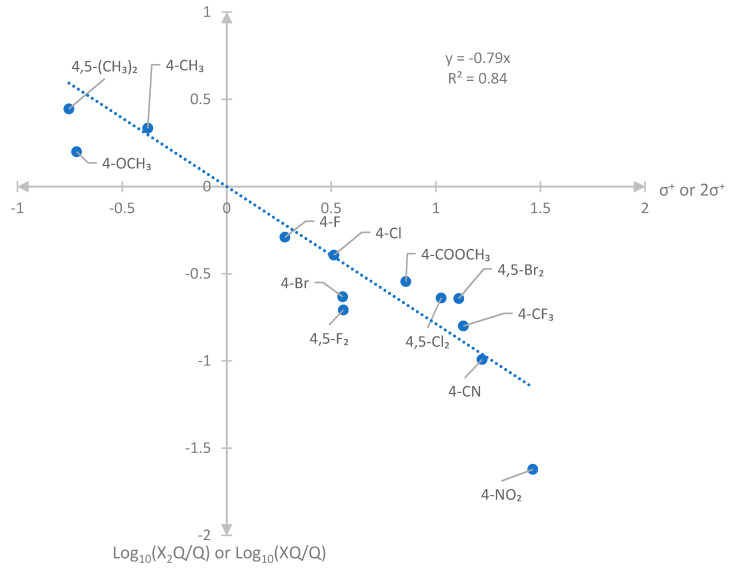
Hammett plot of **XQ/Q** ratio with standard composite σ+ constants and **X_2_Q/Q** ratio with doubled standard composite σ+ constants.

**Figure 5:**
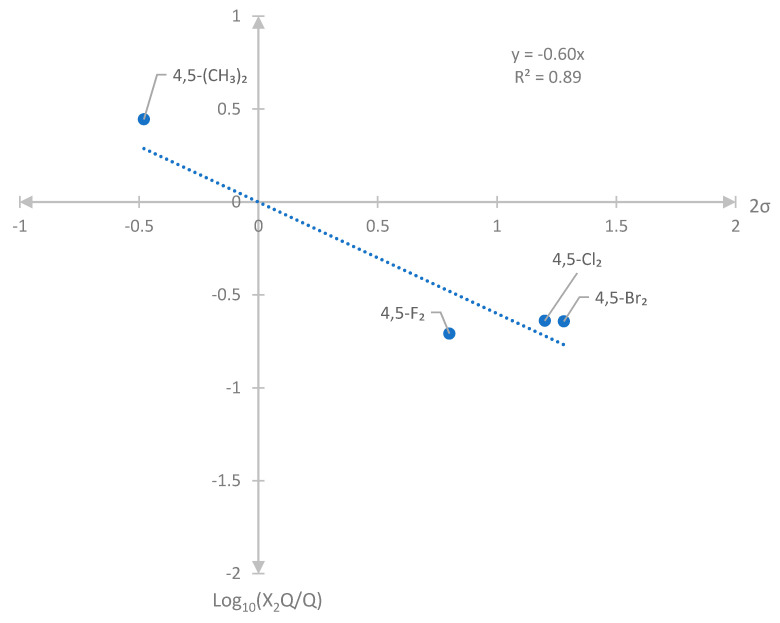
Hammett plot of **X_2_Q/Q** ratio with doubled standard composite σ constants.

**Figure 6:**
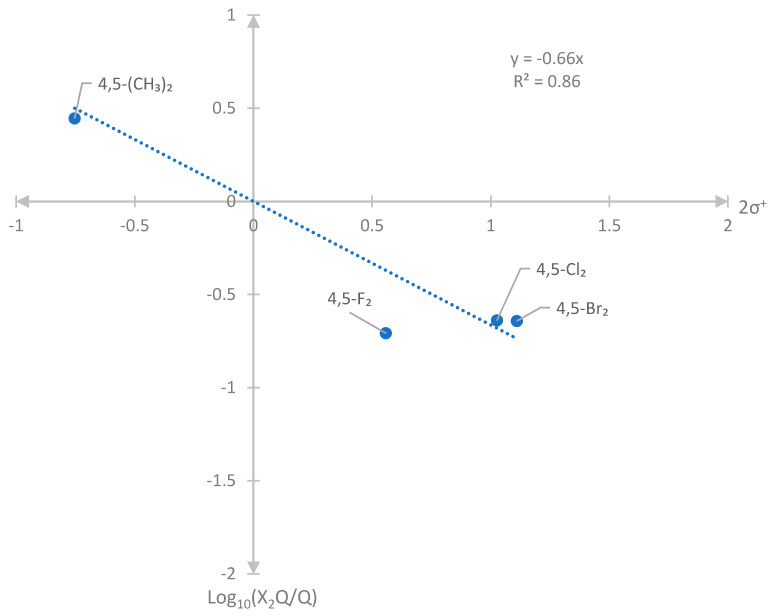
Hammett plot of **X_2_Q/Q** ratio with doubled standard composite σ+ constants.

**Figure 7:**
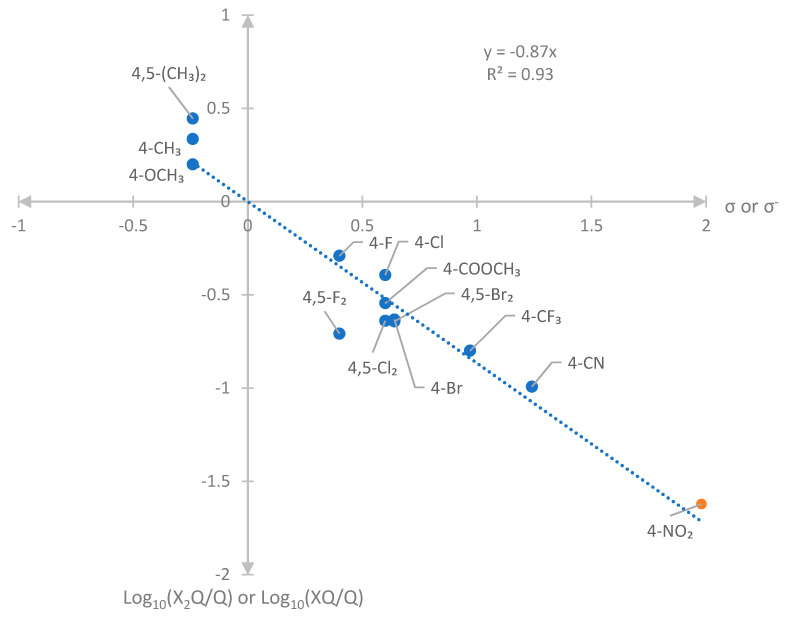
Hammett plot of **XQ/Q** or **X_2_Q/Q** ratio with standard composite σ constants except for X = NO_2_ (σ− constant, highlighted in orange).

**Figure 8:**
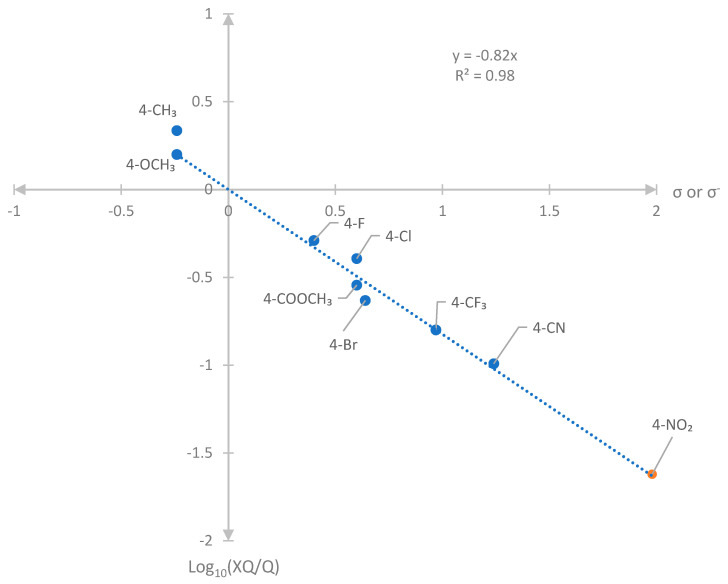
Hammett plot of **XQ/Q** ratio with standard composite σ constants except for X = NO_2_ (σ− constant, highlighted in orange).

These trends in the **XQ/Q** or **X_2_Q/Q** ratio can be interpreted in greater detail by constructing a Hammett correlation. Figure 1, Figure 2, Figure 3, Figure 4, Figure 5, Figure 6, Figure 7 and Figure 8 show the plots that result by considering various methods for assigning constants to the substituent(s). In this discussion, ρ constants and R^2^ values are quoted to two decimal places.

Figure 1 is constructed on the basis of standard composite σ constants. The result is a good correlation, with a ρ value of −0.96 and an R^2^ value of 0.96, which is comparable with that (0.89 or 0.90) obtained in the earlier competition experiments in which **B** and **BY_2_** competed to condense with **P** to form **Q** and **QY_2_** [7]. This good agreement suggests that using standard σ values generally may be appropriate in these analyses.

Figure 2 is constructed using composite σ+ constants. In general, σ+ constants tend to be more appropriate than σ constants in cases in which there is strong stabilizing conjugative interaction (typically by the mesomeric effect) of the substituent with the second group (acting as a nucleophile or electrophile). There is a slight numerical reduction in the ρ value (to a less negative value of −0.89) relative to that derived on the basis of sigma constants; moreover, there is a somewhat larger decline in the R^2^ value (to 0.90).

Figure 3 and Figure 4 show the corresponding graphs that are constructed if the composite σ or σ+ constants are doubled to allow for the presence of two substituents in **X_2_P**. With doubled composite σ constants, ρ is numerically reduced to −0.80, with an R^2^ value of 0.93, which is slightly less than that obtained in Figure 1. When doubled σ+ constants are employed, the numerical value of ρ falls a little further, (to −0.79), and the R^2^ factor is slightly reduced (to 0.91). In each case, the numerical value of ρ is reduced, relative to that when standard composite σ or σ+ constants are used. Moreover, the R^2^ values are also appreciably less impressive when doubled σ or σ+ constants are employed. These factors suggest that there is little, if any, advantage in employing doubled constants to construct Hammett correlations in this system, even though there are qualitative trends in the **X_2_Q/Q** compared to the **XQ/Q** ratio that appear to indicate that the second substituent does have an effect that reinforces that of the first substituent.

The idea that caution is necessary in using doubled σ or σ+ constants is confirmed by considering the Hammett plots of Figure 5 and Figure 6, that are obtained from only the four **X_2_Q/Q** ratios. The derived ρ constant (−0.60 and −0.66, respectively) is significantly reduced, partly because the doubling of the σ or σ+ constants stretches the abscissa), thus suggesting that this procedure may be inappropriate, even if relatively good R^2^ values (0.93 and 0.92, respectively) are obtained. Moreover, the point for X = F is out of line with the other three cases in both graphs, thus suggesting that the approximations in deriving doubled σ or σ+ constants do not always hold good.

Another obvious feature of the best Hammett plot of Figure 1 is that the point for X = NO_2_ lies appreciably out of line with those for the other electron-withdrawing substituents. As noted previously, **O_2_NP** is so deactivated that condensing it with **B** in classical solution experiments to form **O_2_NQ** requires the use of a different solvent and higher temperatures. This greatly reduced reactivity reflects the powerful −M effect of the nitro group. If the σ− constant is used for this substituent to produce the Hammett plot of Figure 7, the R^2^ value is increased to 0.96 and the ρ constant becomes −0.87. These improvements are further enhanced if the problematic doubly substituted cases are excluded, giving a ρ constant of −0.82 and an R^2^ value of 0.99.

Several significant deductions may be made from these Hammett correlations. Firstly, despite variations in the ρ values derived from Figure 1, Figure 2, Figure 3, Figure 4, Figure 5, Figure 6, Figure 7 and Figure 8, it is clear that the electron density on the amino group(s) is reduced during the rate-limiting step in the accelerated condensation that forms **XQ** or **X_2_Q** in the microdroplets. In turn, this finding indicates that **P**, **XP** or **X_2_P** behaves as a nucleophile in the condensation. This result complements the earlier finding that the electron density on the carbonyl group of the benzil increases during the rate-limiting step of the reaction, thus indicating that this component behaves as an electrophile [7].

Secondly, the magnitude of ρ (−0.96 when σ constants are used in Figure 1, or −0.82 if the σ− constant is used only for X = NO_2_, but σ constants are used for all other substituents) reveals that the reduction in electron density on the amino group(s) is considerable, corresponding to approximately half the value of −1.88 for hydrolysis of substituted benzyl chlorides in acetone and water at 69 °C [22]. It is, however, numerically smaller than the value of 1.86 that was found in the previous investigation of the condensation of **P** with **B** and **BY_2_** [7]. Several explanations may be devised for this difference. One obvious, if somewhat speculative, possibility is that there are two amino groups in **P**, **XP** and **X_2_P**, which means that the effect of a substituent will be divided between these groups, thus reducing its effect on either.

Thirdly, despite the complication caused by the presence of two amino groups in **XP**, one of which is para to X, whereas the other is meta to X, a valuable analysis using composite substituent constants is possible. If only one variety of substituent constant is used, the best correlation occurs for standard σ constants, with a good R^2^ value of 0.92. Substituting σ+ constants gives a significantly less good correlation (R^2^ value of 0.82). Similarly, there is little, if any, benefit in using “doubled” substituent constants in constructing Hammett plots in this system. However, there is a case to use the σ− constant for the nitro group, which has a particularly powerful −M effect, in which case an even better correlation (R^2^ value of 0.99) is obtained.

## 4. Materials and Methods

The required **Q**, **XQ** and **X_2_Q** reference samples were prepared from commercial starting materials of high purity by condensation of **P**, **XP** or **X_2_P** with **B**, usually in ethanol at ~80 °C [23] [but in ethylene glycol or glycerol at 120 °C for **O_2_NQ**, which was not formed at an appreciable rate in ethanol]. All these materials had the expected spectroscopic properties; no impurities were detected by GC-MS or other means.

The competition experiments were conducted by a slight modification of the method described previously [7]. In brief, equimolar quantities of **P**, **XP** or **X_2_P** with **B** in freshly prepared solution in methanol were mixed. Two 300 μL portions of the combined solution containing these components were transmitted via a Hamilton syringe through the nebulizer of a Z-spray^TM^ electrospray ionization source of a Waters mass spectrometer. The nebulized spray was collected, taken at once into the syringe, transmitted again through the nebulizer, collected again, and transmitted through the nebulizer a third time, collected and analyzed by injection into the GC-MS system. This triple transmission through the nebulizer gave a conversion (usually 30–70%) that facilitated analysis of the resultant mixture of products. As noted in previous work, the reactions in the microdroplets in the nebulizer at room temperature took place much more quickly than the classical solution syntheses (which usually required 1–5 h at 80 °C). Condensation of the components before or after transmission through the nebulizer was shown to be insignificant by control experiments in which mixtures of **P**, **XP** or **X_2_P** with **B** were allowed to stand in solution at room temperature.

During the initial exploratory work, the possibility of performing four component experiments was investigated, but found to entail practical difficulties. In response to the suggestion of a reviewer, a mixture of equimolar solutions of **P**, **(CH_3_)_2_P**, **B** and **B4,4′F_2_** was transmitted through the nebulizer as described above. The chromatogram and associated peak areas (Figure S14 and Table S14) were satisfactory in this case; however, the increased possibility of peak overlap and the greater difficulty in interpreting the data make this otherwise attractive option less reliable than doing a series of three component reactions.

The GC-MS analysis was performed on a 7890 gas chromatograph attached to a 5975 EI Inert MSD (Agilent Technologies, Santa Clara, CA, USA) operating at 70 eV; the temperature of the source and quadrupole was 230 and 150 °C, respectively. Data were acquired over the *m*/*z* range 50–450. Samples of the solution obtained after transmission through the nebulizer were admitted to a split/splitless inlet held at 300 °C with a 2:1 split. Chromatographic separation of components was achieved in a 30 m × 0.25 mm 5% diphenyl low-polarity fused-silica capillary column, using helium as the carrier gas at a flow rate of 1.2 mL min^−1^. The initial temperature was 100 °C, increasing linearly at 25 °C minute^−1^ to 350 °C, where it was maintained for 2 min.

## 5. Conclusions

Competition experiments permit the development of a useful structure–reactivity relationship in the accelerated formation in microdroplets of substituted 2,3-diphenylquinoxalines from substituted phenylenediamines. Despite complications caused by the presence of two amino groups in one component, a good Hammett correlation is possible, revealing that the phenylenediamine behaves as a nucleophile in this condensation. The use of standard composite substituent constants appears to give the best correlation, but using the σ- constant may be justifiable when the substituent is a nitro group. The successful construction of a valuable Hammett correlation in this accelerated condensation indicates that devising and implementing appropriate competition experiments offers a rapid, reproducible and effective protocol for studying the reaction kinetics of these interesting processes. Moreover, insight into the mechanism of these accelerated reactions may be conveniently obtained by this approach.

## Figures and Tables

**Table 1:** Ratio of 2,3-diphenylquinoxaline and 6-substituted-2,3-diphenylquinoxaline formed in competition experiments in the nebulizer.

Reagents ^1^			Products ^2^		Ratio (XQ/Q) ^3^
**P**	**FP**	**B**	**Q**	**FQ**	0.512
**P**	**ClP**	**B**	**Q**	**ClQ**	0.404
**P**	**BrP**	**B**	**Q**	**BrQ**	0.233
**P**	**CH_3_P**	**B**	**Q**	**CH_3_Q**	2.16
**P**	**CH_3_OP**	**B**	**Q**	**CH_3_OQ**	1.58
**P**	**CH_3_O_2_CP**	**B**	**Q**	**CH_3_O_2_CQ**	0.228
**P**	**CF_3_P**	**B**	**Q**	**CF_3_Q**	0.159
**P**	**NCP**	**B**	**Q**	**NCQ**	0.102
**P**	**O_2_NP**	**B**	**Q**	**O_2_NQ**	0.024

^1^ Equimolar quantities of **P**, **XP** and **B** in methanol infused through the nebulizer. ^2^ Products 2,3-diphenylquinoxaline, **Q**, and 6-substituted-2,3-diphenylquinoxaline, **XQ**. ^3^ Ratio of products determined by the relative areas of the peaks in the TIC from GC-MS; the quoted values are derived from several independent measurements. Most values are quoted to three significant figures only to avoid introducing rounding errors. GC-MS traces and peak areas are available in the Supplementary Materials.

**Table 2:** Ratio of 2,3-diphenylquinoxaline and 6,7-disubstituted-2,3-diphenylquinoxaline formed in competition experiments in the nebulizer.

Reagents ^1^			Products ^2^		Ratio (X_2_Q/Q) ^3^
**P**	**F_2_P**	**B**	**Q**	**F_2_Q**	0.196
**P**	**Cl_2_P**	**B**	**Q**	**Cl_2_Q**	0.229
**P**	**Br_2_P**	**B**	**Q**	**Br_2_Q**	0.228
**P**	**(CH_3_)_2_P**	**B**	**Q**	**(CH_3_)_2_Q**	2.79

^1^ Equimolar quantities of **P**, **X_2_P** and **B** in methanol infused through the nebulizer. ^2^ Products 2,3-diphenylquinoxaline, **Q**, and 6,7-disubstituted-2,3-diphenylquinoxaline, **X_2_Q**. ^3^ Ratio of products determined by the relative areas of the peaks in the TIC from GC-MS; the quoted values are derived from several independent measurements. Most values are quoted to three significant figures only to avoid introducing rounding errors. GC-MS traces and peak areas are available in the Supplementary Materials.

## Supplementary Materials

The following are available online, Figure S1: Chromatogram for competition of **P**, **B** and **FP**; Table S1: Peak information for competition of **P**, **B** and **FP**; Figure S2: Chromatogram for competition of **P**, **B** and **F_2_P**; Table S2: Peak information for competition of **P**, **B** and **F_2_P**; Figure S3: Chromatogram for competition of **P**, **B** and **ClP**; Table S3: Peak information for competition of **P**, **B** and **ClP**; Figure S4: Chromatogram for competition of **P**, **B** and **Cl_2_P**; Table S4: Peak information for competition of **P**, **B** and **Cl_2_P**; Figure S5: Chromatogram for competition of **P**, **B** and **BrP**; Table S5: Peak information for competition of **P**, **B** and **BrP**; Figure S6: Chromatogram for competition of **P**, **B** and **Br_2_P**; Table S6: Peak information for competition of **P**, **B** and **Br_2_P**; Figure S7: Chromatogram for competition of **P**, **B** and **CH_3_P**; Table S7: Peak information for competition of **P**, **B** and **CH_3_P**; Figure S8: Chromatogram for competition of **P**, **B** and **(CH_3_)_2_P**; Table S8: Peak information for competition of **P**, **B** and **(CH_3_)_2_P**; Figure S9: Chromatogram for competition of **P**, **B** and **CH_3_OP**; Figure S9: Chromatogram for competition of **P**, **B** and **CH_3_OP**; Figure S10: Chromatogram for competition of **P**, **B** and **CF_3_P**; Table S10: Peak information for competition of **P**, **B** and **CF_3_P**; Figure S11: Chromatogram for competition of **P**, **B** and **CH_3_O_2_CP**; Table S11: Peak information for competition of **P**, **B** and **CH_3_O_2_CP**; Figure S12: Chromatogram for competition of **P**, **B** and **NCP**; Table S12: Peak information for competition of **P**, **B** and **NCP**; Figure S13: Chromatogram for competition of **P**, **B** and **NO_2_P**; Table S13: Peak information for competition of **P**, **B** and **NO_2_P**; Figure S14: Chromatogram for competition of **P**, **(CH_3_)_2_P**, **B** and **B4,4′F_2_**; Table S14: Peak information for competition of **P**, **(CH_3_)_2_P**, **B**, **B4,4′F_2_**.

## Sample Availability

Samples of the authentic compounds (**Q**, **FQ**, **F_2_Q**, **ClQ**, **Cl_2_Q**, **BrQ**, **Br_2_Q**, **CH_3_Q**, **[CH_3_]_2_Q**, **CH_3_OQ**, **CF_3_Q**, **CH_3_O_2_CQ**, **NCQ**, **NO_2_Q**) are available from the authors.
