# Supplementary material for: Hammett Correlation in the Accelerated Formation of 2,3-Diphenylquinoxalines in Nebulizer Microdroplets
Source: Molecules. 2021 Aug 21;26(16):5077. doi: 10.3390/molecules26165077 (PMC8399188; doi:10.3390/molecules26165077)
Supplement: Supplementary file 1 [file molecules-26-05077-s001.zip › molecules-1290975-SI.pdf]

Supplementary Materials

Peaks labelled in chromatograms are for the quinoxaline products of the reaction, with peak areas given in the subsequent table. Other peaks of significant intensity are from unreacted starting materials. Abbreviations used are explained in the main text.

Figure S1: Chromatogram for competition of P, B and FP

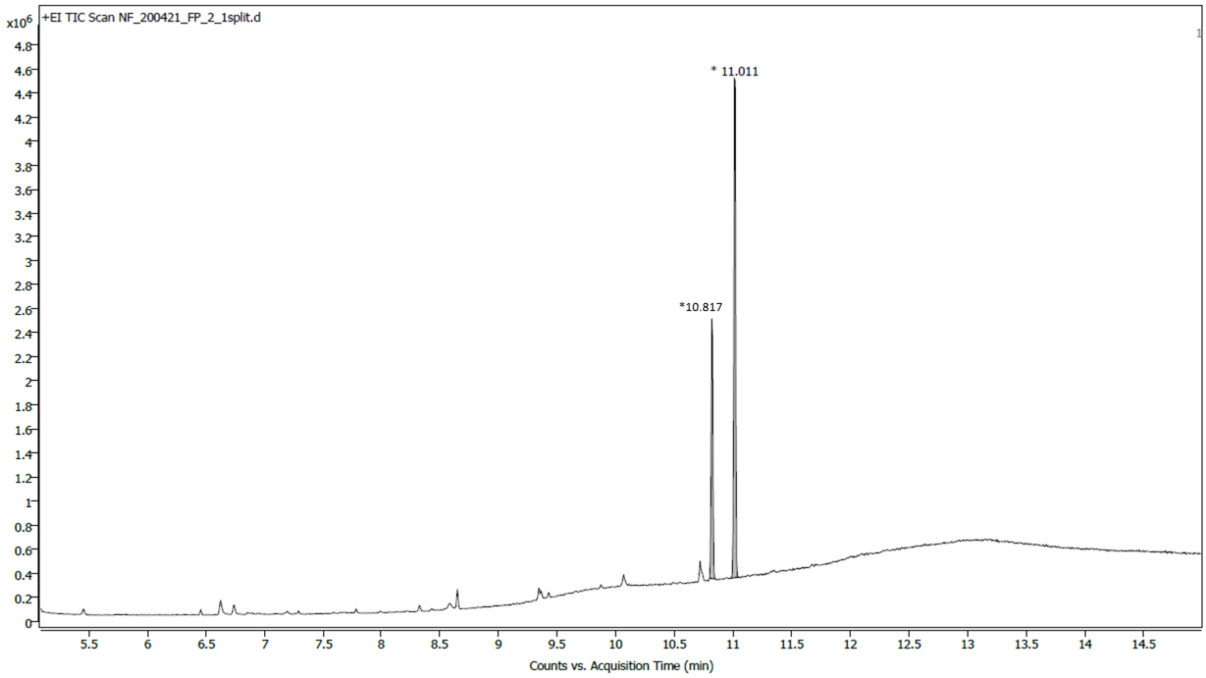

Table S1: Peak information for competition of P, B and FP

Retention Time (minutes)	Compound	Peak Area
10.817	FQ	2278456
11.011	Q	4449338

Figure S2: Chromatogram for competition of P, B and F_2P

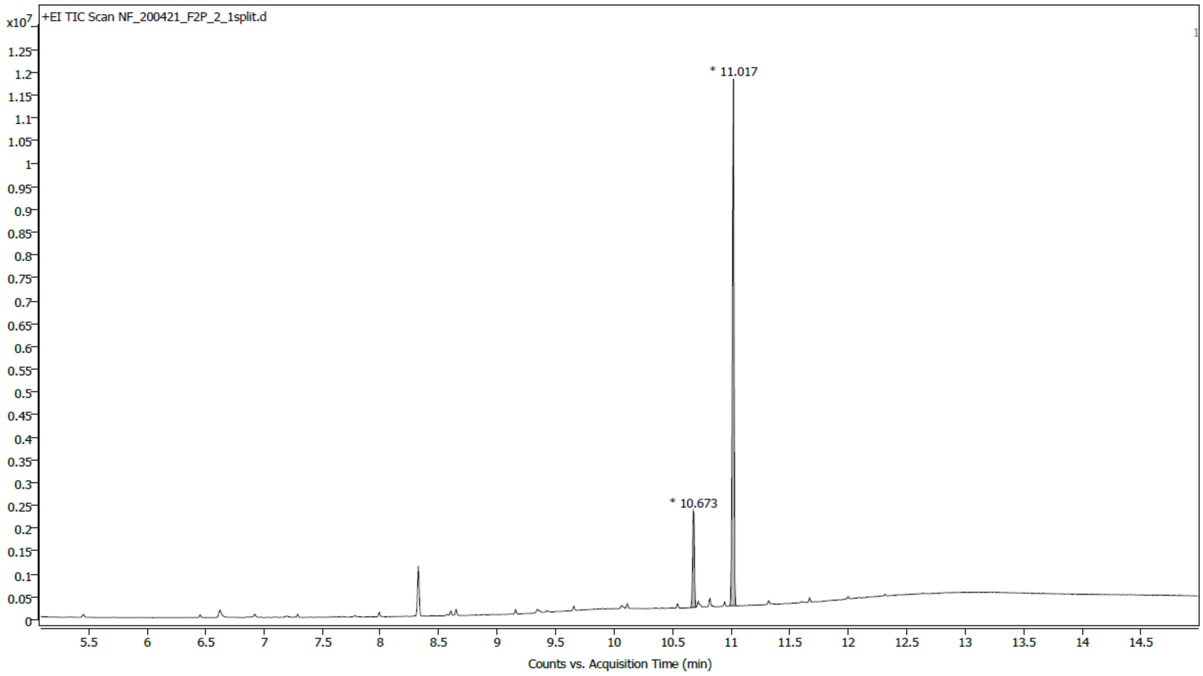

Table S2: Peak information for competition of P, B and F_2P

Retention Time (minutes)	Compound	Peak Area
10.673	F_2Q	2249887
11.017	Q	11483882

Figure S3: Chromatogram for competition of P, B and ClP

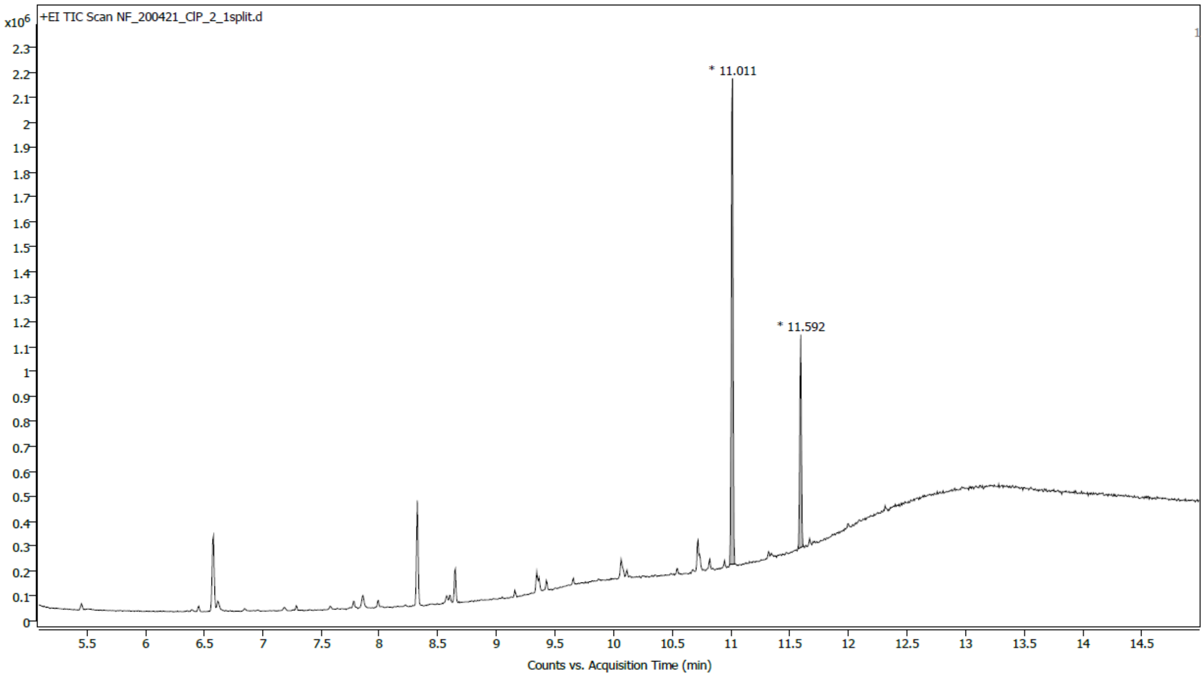

Table S3: Peak information for competition of P, B and ClP

Retention Time (minutes)	Compound	Peak Area
11.011	Q	2071985
11.592	ClQ	837054

Figure S4: Chromatogram for competition of P, B and Cl_2P

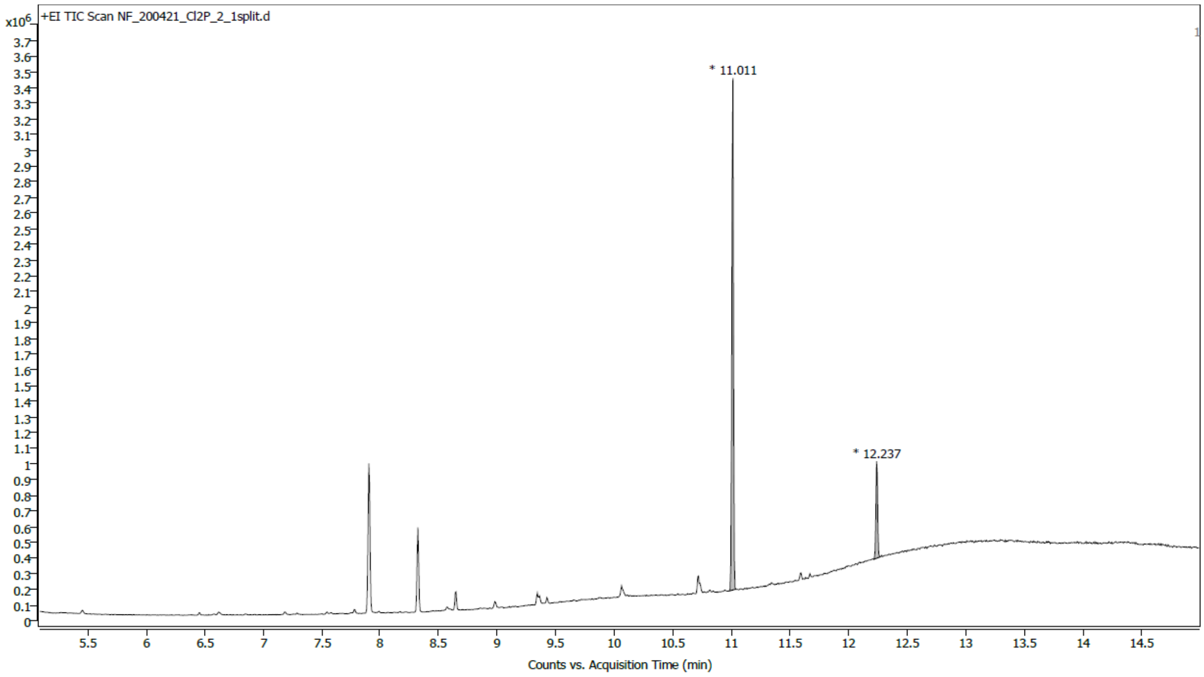

Table S4: Peak information for competition of P, B and Cl_2P

Retention Time (minutes)	Compound	Peak Area
11.011	Q	3199273
12.237	Cl_2Q	734213

Figure S5: Chromatogram for competition of P, B and BrP

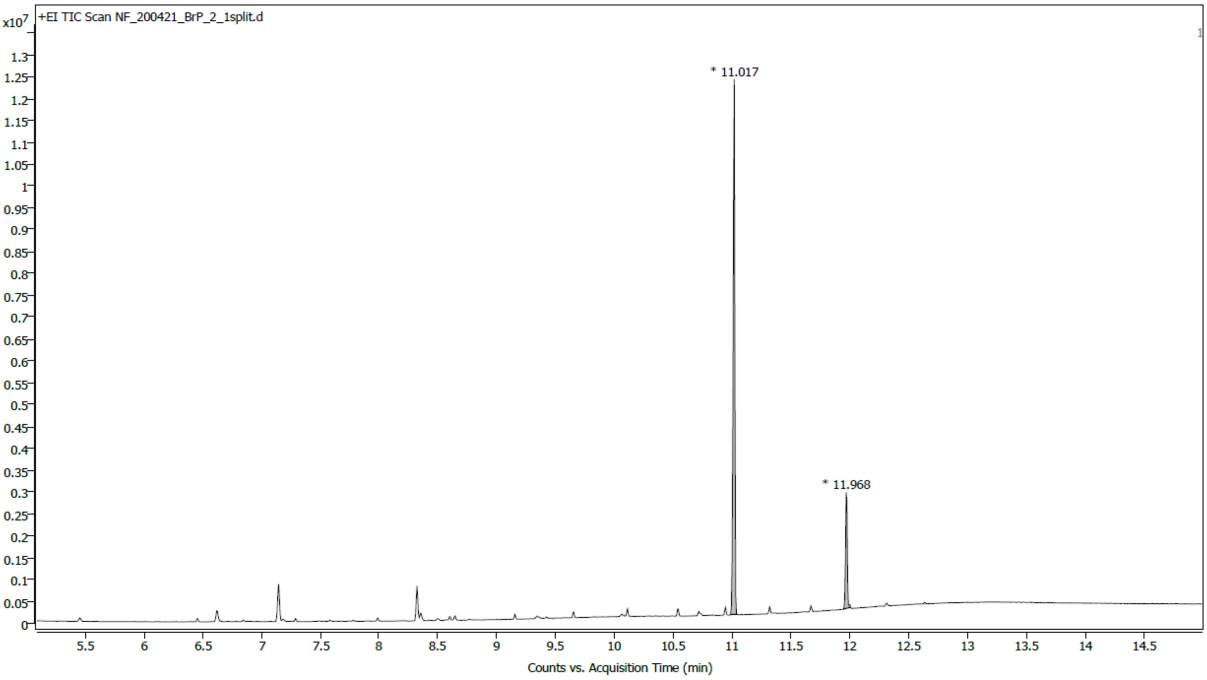

Table S5: Peak information for competition of P, B and BrP

Retention Time (minutes)	Compound	Peak Area
11.017	Q	12157642
11.968	BrQ	2835429

Figure S6: Chromatogram for competition of P, B and Br_2P

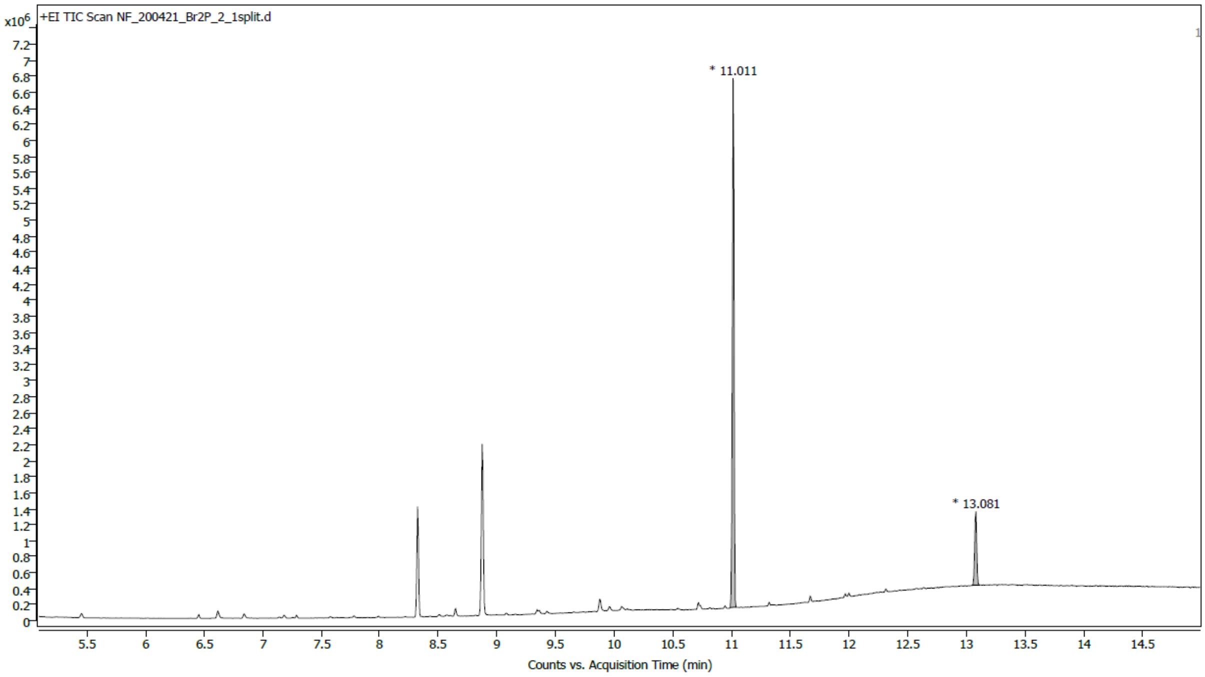

Table S6: Peak information for competition of P, B and Br_2P

Retention Time (minutes)	Compound	Peak Area
11.011	Q	6719543
13.081	Br_2Q	1531152

Figure S7: Chromatogram for competition of P, B and CH_3P

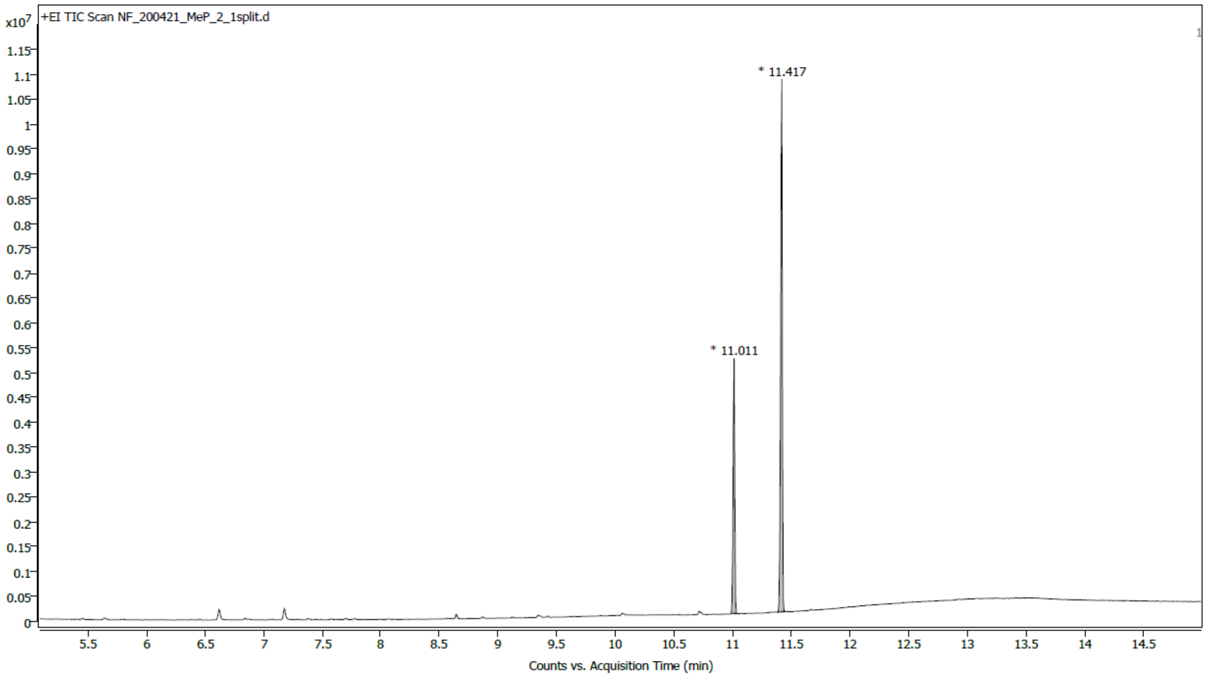

Table S7: Peak information for competition of P, B and CH_3P

Retention Time (minutes)	Compound	Peak Area
11.011	Q	4952474
11.417	CH_3Q	10712490

Figure S8: Chromatogram for competition of P, B and $(CH_3)_2P$

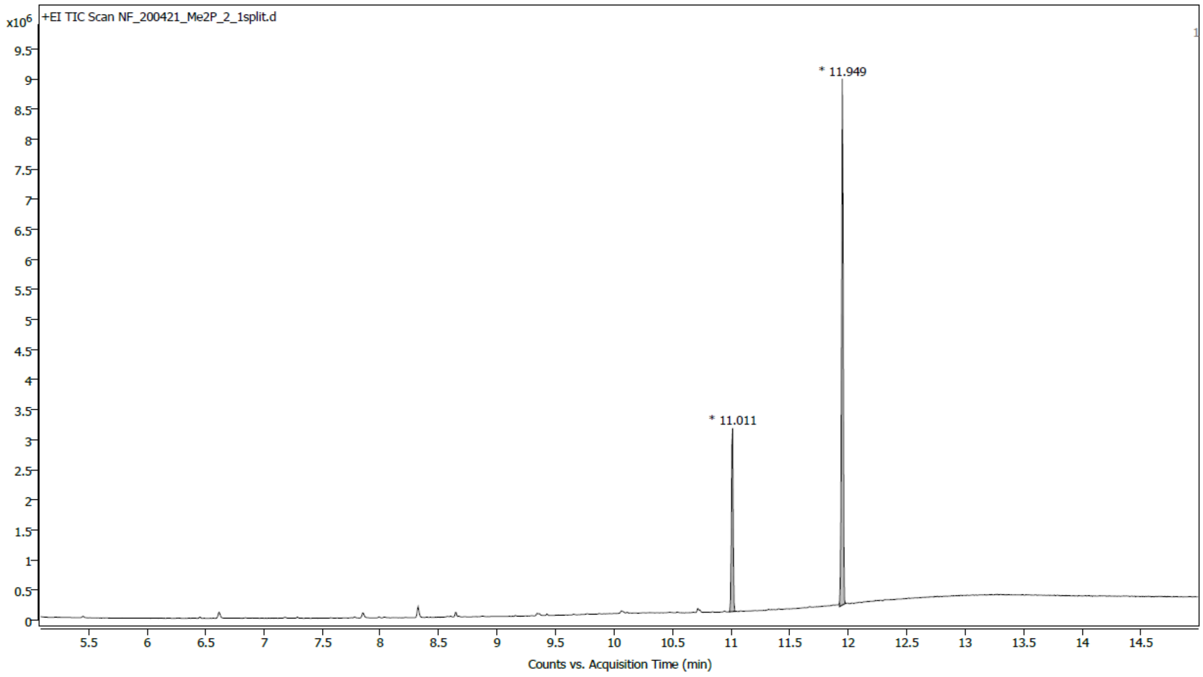

Table S8: Peak information for competition of P, B and $(CH_3)_2P$

Retention Time (minutes)	Compound	Peak Area
11.011	Q	3130334
11.949	$(CH_3)_2Q$	8726454

Figure S9: Chromatogram for competition of P, B and CH_3OP

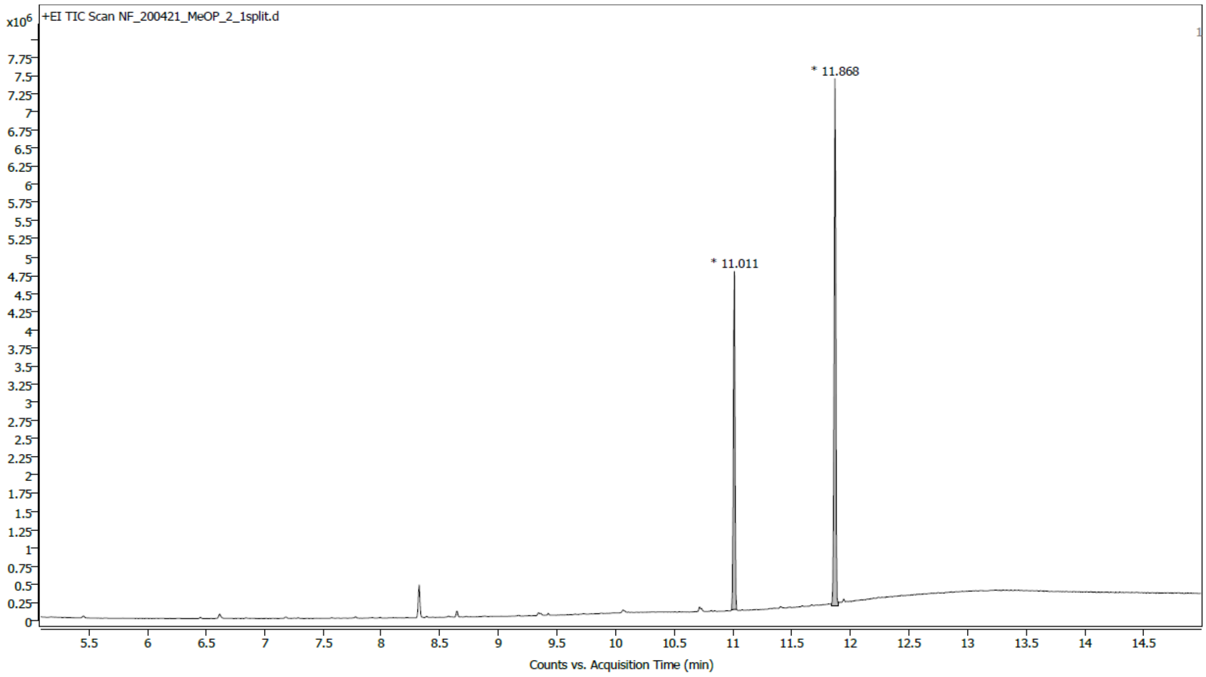

Figure S9: Chromatogram for competition of P, B and CH_3OP

Retention Time (minutes)	Compound	Peak Area
11.011	Q	4565609
11.868	CH_3OQ	7223694

Figure S10: Chromatogram for competition of P, B and CF_3P

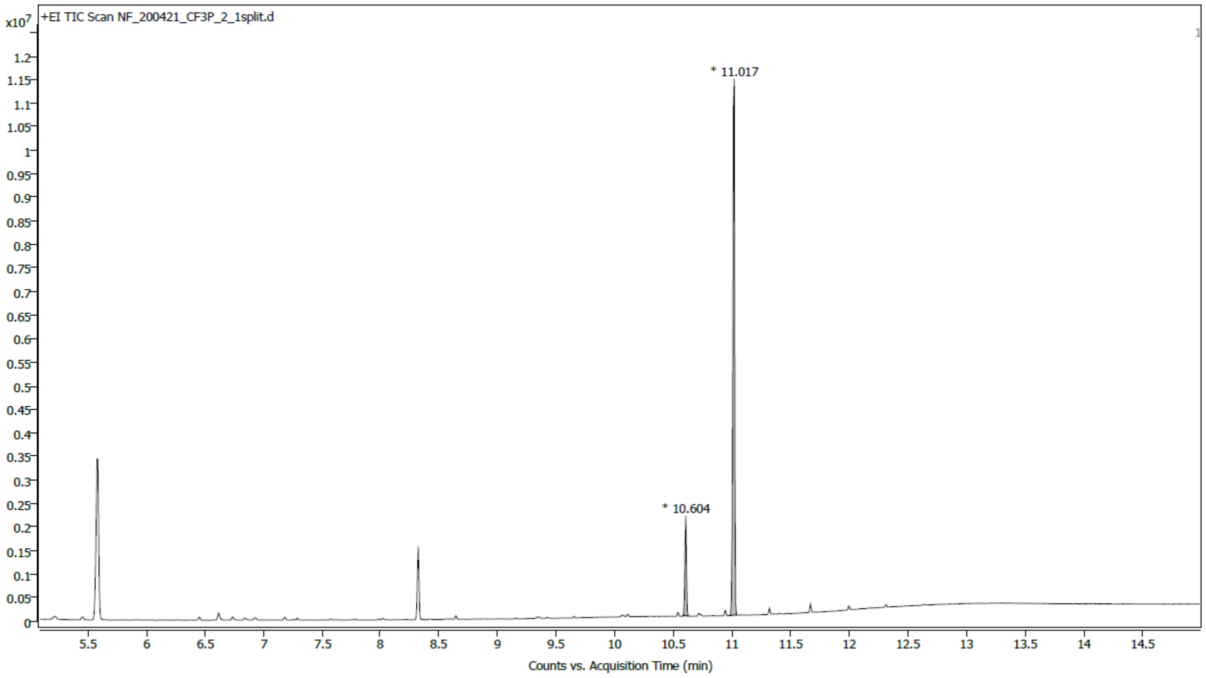

Table S10: Peak information for competition of P, B and CF_3P

Retention Time (minutes)	Compound	Peak Area
10.604	CF_3Q	1926036
11.017	Q	12138765

Figure S11: Chromatogram for competition of P, B and CH_3OOCP

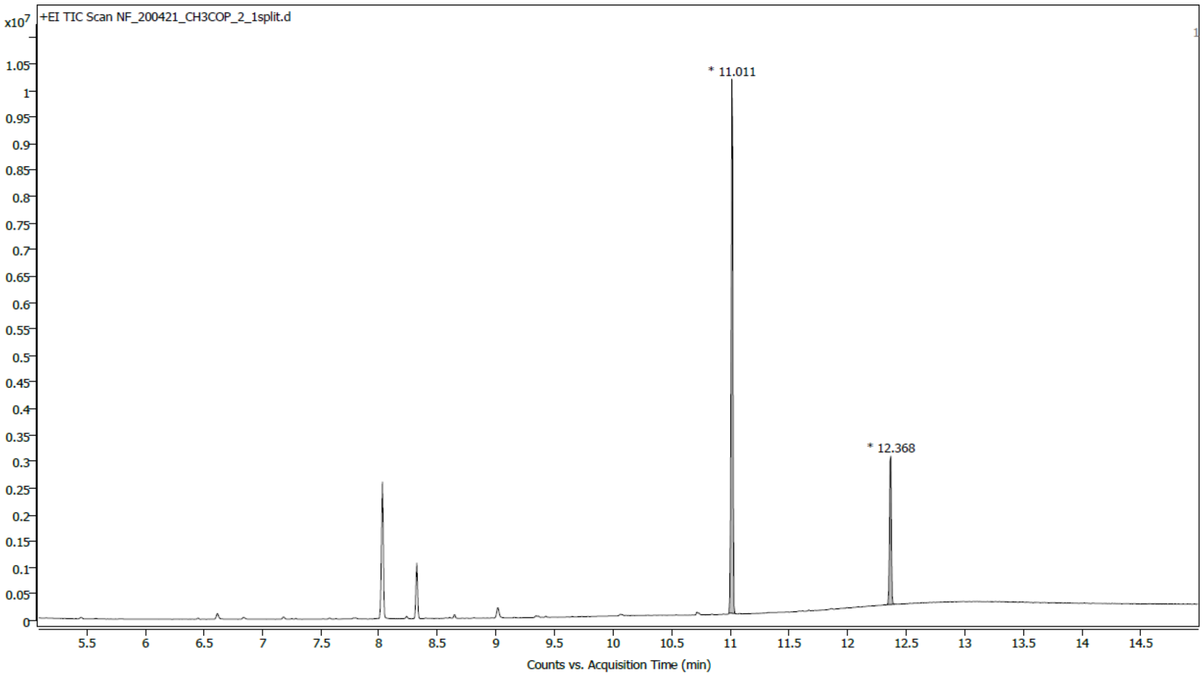

Table S11: Peak information for competition of P, B and CH_3OOCP

Retention Time (minutes)	Compound	Peak Area
11.011	Q	10763380
12.368	CH_3OOCQ	3070028

Figure S12: Chromatogram for competition of P, B and NCP

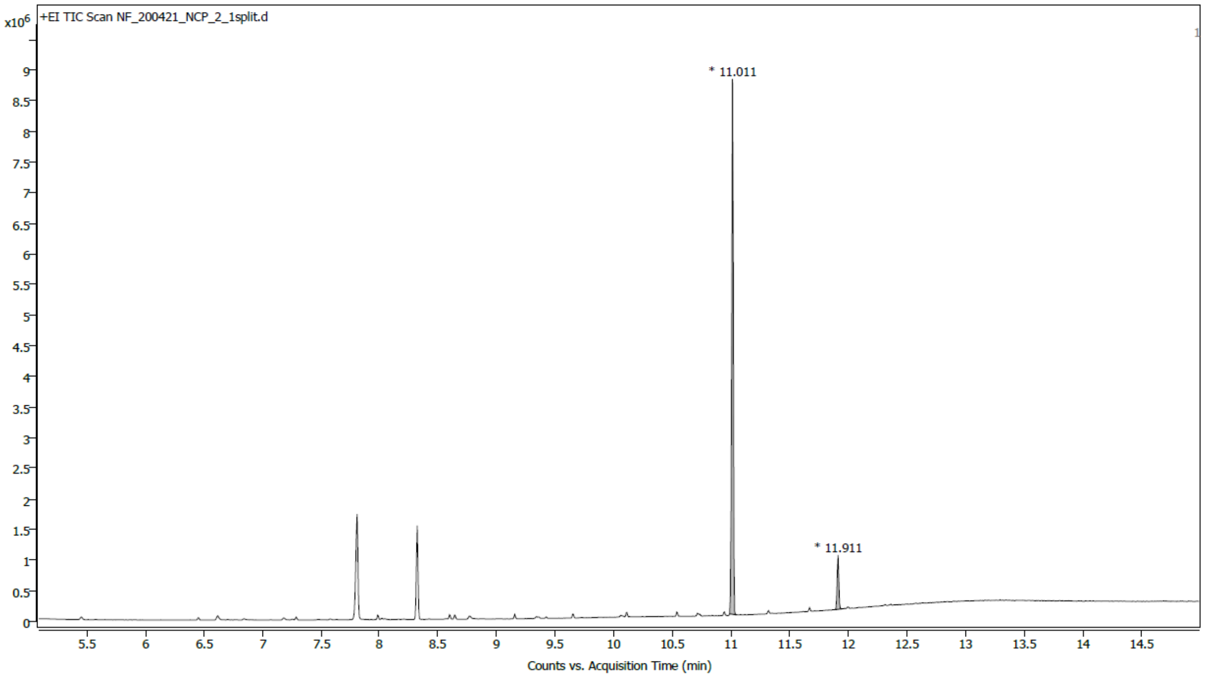

Table S12: Peak information for competition of P, B and NCP

Retention Time (minutes)	Compound	Peak Area
11.011	Q	8425667
11.911	NCQ	858243

Figure S13: Chromatogram for competition of P, B and NO_2P

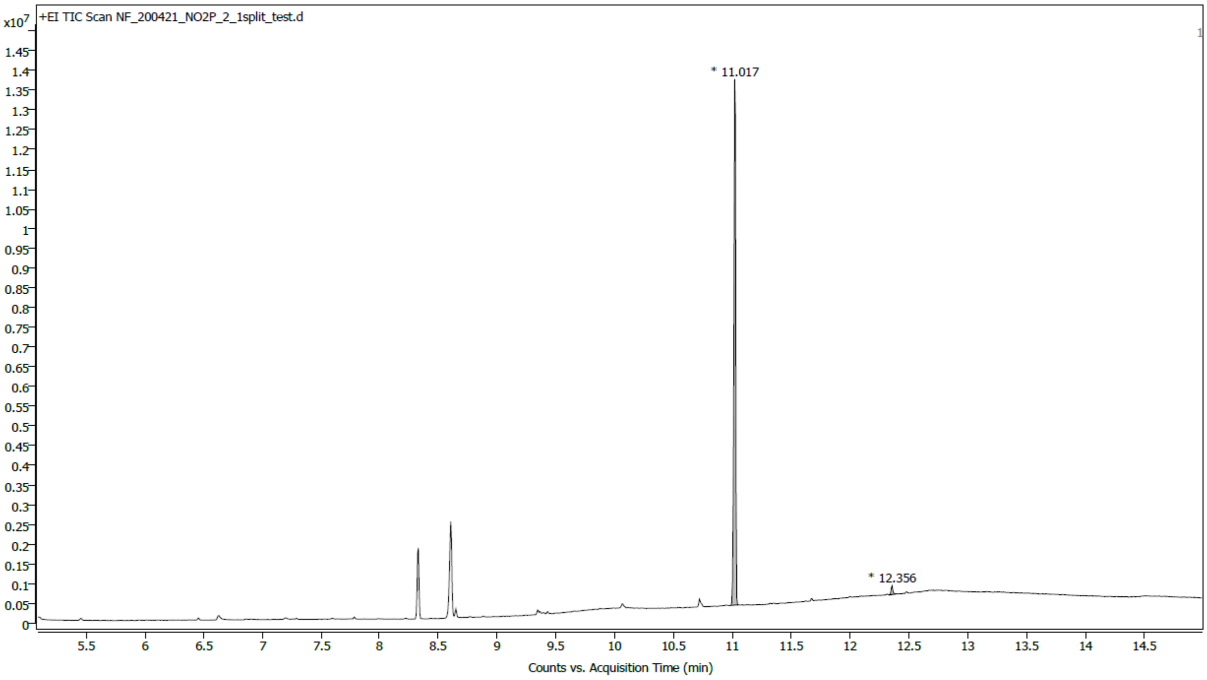

Table S13: Peak information for competition of P, B and NO_2P

Retention Time (minutes)	Compound	Peak Area
11.017	Q	14687656
12.356	NO_2Q	350914
